# Does chlorhexidine reduce bacteremia following tooth extraction? A systematic review and meta-analysis

**DOI:** 10.1371/journal.pone.0195592

**Published:** 2018-04-23

**Authors:** Iciar Arteagoitia, Carlos Rodriguez Andrés, Eva Ramos

**Affiliations:** 1 Department of Stomatology I, University of the Basque Country (UPV/EHU) Bizkaia, Spain; 2 BioCruces Health Research Institute, Bizkaia, Spain; 3 Department of Epidemiology and Public Health, University of the Basque Country (UPV/EHU) Bizkaia, Spain; Universidade de Sao Paulo, BRAZIL

## Abstract

**Background and aims:**

Scientific evidence is not clear regarding the use of antimicrobial mouth rinse before dental extraction to reduce bacteremia. We tested the null hypothesis that there would be no difference in the incidence of bacteremia following dental extractions in patients treated with or without chlorhexidine.

**Material and methods:**

We conducted a meta-analysis following the recommendations proposed by PRISMA Preferred Reporting Items for Systematic Reviews and Meta-Analyses. The data sources Pubmed, Cochrane, Web of Science, Science Direct, Scopus, and Ovid MD were searched until April 30, 2017. (chlorhexidine) AND (bacteremia OR bacteraemia) AND (extraction OR removal) were used as key words in a free-text search. Published meeting abstracts were searched. The references of each article were reviewed. We only included randomized controlled clinical trials. There were no restrictions regarding language or date of publication. The outcome measure was the incidence of the bacteremia measured within the first ten minutes post-extraction. Two reviewers independently undertook the risk of bias assessment and data extraction. A fixed-effects inverse variance weighted meta-analysis was conducted.

**Results:**

Out of 18 studies, eight eligible trials with 523 participants were selected, 267 in the experimental group and 256 in the control group: risk ratio = 0.882 (95% confidence interval 0.799 to 0.975; p = 0.014), heterogeneity I^2^ = 13.07%, and p = 0.33. The number needed to treat was 16 (95% CI 7-Infinity).

**Conclusions:**

Approximately 12% of bacteremia cases can be prevented if a population is exposed to chlorhexidine. CRD42016046586.

## Introduction

Tooth extractions almost always cause bacteremia due to the large presence of bacteria in the oral cavity [1]. The importance of this transient bacteremia in the pathogenesis of infectious endocarditis (IE) and prosthesis infection and whether it contributes to local infection after oral surgeries [2] are controversial.

Studies have shown that chlorhexidine mouthwash has a strong antimicrobial effect on saliva microflora [3,4] and supragingival plaque [5]. For these reasons, one should assume that antimicrobial mouthwashes used by the patient before a dental procedure should decrease the number of microorganisms introduced into the patient’s bloodstream. However, the scientific evidence is not clear. The American Heart Association (AHA) in 1997 [6] suggested that patients at risk for IE should use an antimicrobial mouthwash before dental treatment. In 2006, the British Society for Antimicrobial Chemotherapy (BSAC) [7] recommended a single mouthwash with 0.2% chlorhexidine (CHX) (10 ml for 1 minute) before dental procedures associated with bacteremia in patients at risk of IE. However, in 2007, the AHA [8] recommended against the use of any antiseptic prophylaxis protocol. The National Institute for Health and Care Excellence [9] (2008) recommended that chlorhexidine mouthwash should not be offered as prophylaxis against IE to people at risk of IE undergoing dental procedures.

Chlorhexidine has been the most widely researched antiseptic for the prevention of bacteremia after dental manipulations, although contradictory results have been reported in the literature [10, 11].

We conducted a systematic review and meta-analysis of randomized controlled trials (RCTs) to evaluate the efficacy of chlorhexidine administered pre-dental extraction to prevent bacteremia. We tested the null hypothesis that there was no difference in bacteremia following dental extractions in patients being treated with or without chlorhexidine.

## Materials and methods

The research was conducted and reported in accordance with the recommendations in the Preferred Reporting Items for Systematic Reviews and Meta-Analyses (PRISMA) statement [12].

The terms searched were descriptors per the Patient, Intervention, Comparison and Outcome (PICO) component.

### Protocol and registration

The review registration information is available in the PROSPERO international prospective register of systematic reviews. https://www.crd.york.ac.uk/prospero/register_new_review.asp

The registration number is CRD42016046586.

Public repository dx.doi.org/10.17504/protocols.io.m65c9g6

### Eligibility criteria

We selected studies that included patients who were adults over 18 years old and of any gender who underwent the extraction of any tooth. For intervention, we included trials that analyzed the efficacy of chlorhexidine mouthwash at any dose or regime. For comparisons, we exclusively included randomized controlled clinical trials (RCTs) without excluding those with split-mouth designs. The outcome was the incidence of bacteremia within the first ten minutes after extraction. For the results of interest, the search was not restricted by language. The bibliographic search began on March 2, 2015, and ended in all databases on April 30, 2017.

### Information sources

The electronic databases consulted were Medline/PubMed, Scopus, ScienceDirect, Web of Science, Evidence-Based Dentistry, ClinicalTrials.gov, the EU Clinical Trials Register, Cochrane Central Register of Controlled Trials, Spanish General University Board database of doctoral theses in Spain (TESEO) and Spanish National Research Council (CSIC) bibliographic databases.

### Search

The search terms selected were descriptors of each PICO component: extraction; removal; tooth; chlorhexidine; and bacteremia. The following filters were applied: humans, clinical trials, meta-analysis, and randomized controlled trials. The electronic search in the Medline/PubMed database was done using MeSH strings and search algorithms connected with Boolean operators as keywords for titles and abstracts. Specifically, we used the following search strategy: *(randomized controlled trials OR controlled****clinical trial***OR randomized controlled trials OR random allocation OR double blind method OR single blind method OR clinical trial OR clinical trials OR (“clinical trial”) OR ((singl* OR doubl* OR trebl* OR tripl*) AND (mask* OR blind*)) OR (“latin square”) OR placebos OR placebo* OR random* OR research design OR comparative study OR evaluation studies OR follow-up studies OR prospective studies OR cross-over studies OR control* OR prospectiv* OR volunteer*) NOT animal AND (chlorhexidine) AND (bacteremia OR bacteraemia) AND (extraction OR removal).

We reviewed the references of all papers retrieved. We searched for conference abstracts, and when potentially unpublished work was identified, we contacted the corresponding authors to request a copy of the study report.

### Study selection

Two researchers performed the database searches separately using the aforementioned criteria. The titles and abstracts of all reports identified were read separately by the two authors. The full report was obtained for those studies that appeared to meet the inclusion criteria or for those in which there was insufficient information in the title or abstract to make a decision. A third researcher was consulted in the case of disagreements. A total of 102 records remained after duplicates were removed. Eighty-four were discarded as not relevant based on title and abstract. Eighteen full-text articles were assessed for eligibility. Ten were excluded for the following reasons: the article referenced another article, the incidence of bacteremia after extraction was not the outcome of interest, the study was carried out only in children, and the most frequent reason was that the study was not a randomized clinical trial.

### Data collection

The selected studies were examined separately by two researchers, both of whom extracted data from each paper. When explicit data on some variables were not stated in the text, they were calculated using data from tables when possible.

### Data items

The following data were extracted for each study included in the meta-analysis: primary author, year of publication, location, recruitment period, number of patients randomized to each study group, details of the surgical procedure and the randomization method, time of collection of blood samples for culture, antiseptic used (including the concentration and dosage form) and the regimen ((dose and guideline), of administration of the antiseptic in each study group, the most bacteria most often identified in the cultures, the incidence of bacteremia according to the time of assessment and the bacteremia data selected for our meta-analysis. Details about adverse reactions, exclusions, withdrawals and losses were also recorded.

### Risk of bias in individual studies

The quality of the clinical trials was assessed via the Oxford Quality Scale [13].

### Summary measures

The efficacy of the treatment was assessed using the relative risk (RR) and preventable fraction. The preventable fraction indicated the percentage of bacteremia cases that would be avoided by treating the control group with chlorhexidine. The effectiveness of the treatment with chlorhexidine was assessed via the number needed to treat (NNT) and number needed to harm (NNH).

### Synthesis of results

All analyses were carried out with StataCorp 2011 Stata Statistical Software: Release 12 (College Station, TX, USA: StataCorp LP). We assessed the heterogeneity of the different studies using the I^2^ test. The overall RR resulting from combining different studies was calculated with a fixed-effects model with weights calculated using the inverse of variance method, and the Mantel-Haenszel method.

### Risk of bias across the studies

The publication bias was assessed graphically using a funnel plot and quantitatively using the methods described by Begg, Egger, Macaskill and Rosenthal.[12]

### Additional analyses

We also carried out a cumulative meta-analysis by publication date, an analysis of influence and an analysis of adverse reactions.

## Results

### Study selection

**Fig 1:**
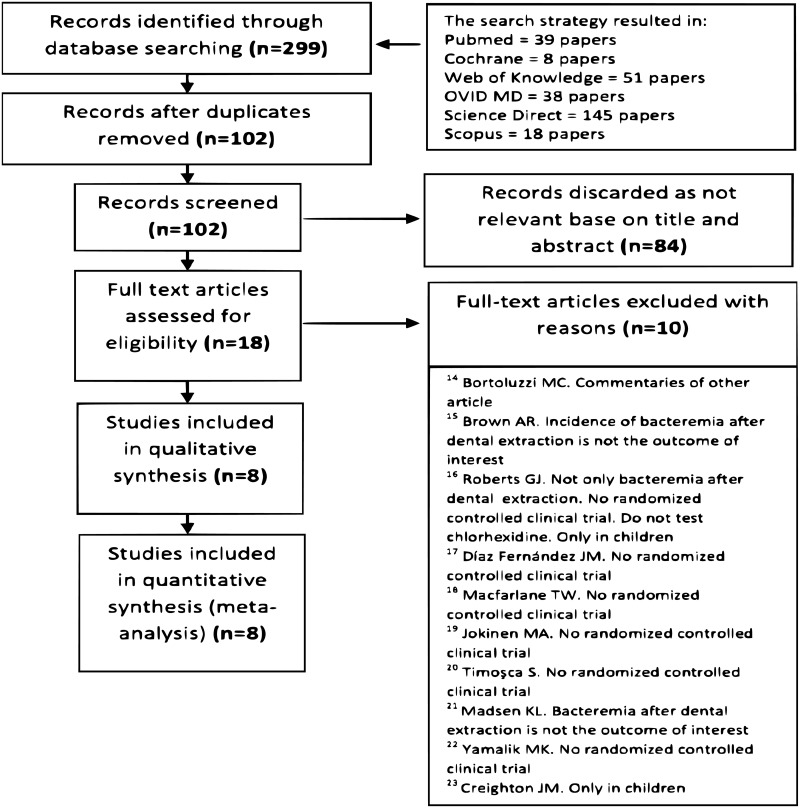
PRISMA flow diagram. Flow diagram showing the study identification, screening and inclusion process.

The study selection process is summarized in “Fig 1”. Ten full text articles were excluded with reasons [14–23].

### Study characteristics

The detailed data of the 8 studies included in the meta-analysis [10, 11, 24–29] are listed in Table 1.

**Table 1:** Characteristics of studies.

Primary author (Year) Location Recruitment period	Study groups	Dental extraction	Surgical procedure	Randomization method	Collection of blood samples for culture	Antiseptic used	Most frequently identified bacteria	Regimens	Patients (%) with positive cultures	Bacteremia values selected for our meta-analysis *	Exclusions, withdrawals and losses	Adverse reactions
Rechmann[29](1989) Germany Not specified	CG (n = 16): NaCl solution-rinse group. EG (n = 17): chlorhexidine group	Only one tooth per patient (23 molars, 8 premolars and 2 front teeth)	All extractions were performed by the same person	Not specified	At baseline (before the start of surgery), 2 minutes and 10 minutes after the extraction	0.1% chlorhexidine mouthwash	Streptococcus	**CG**: Rinsed the mouth with NaCl physiological saline solution for 2 minutes. **EG**: Rinsed the mouth with 0.1% chlorhexidine solution for 2 minutes. Patients received 100 ml of washing solution and were instructed to rinse intensely in the area	Overall:CG: 62.5%. EG: 82.4%	CG: 62.5%EG: 82.4%Values from positive cultures in at least one of the two time points (2 and 10 minutes after the extraction) in which blood samples are collected	-	Not recorded
Lockhart[28] (1996). USA. Not specified	CG (n = 33): placebo-rinse group. EG (n = 37): chlorhexidine group	A single-tooth extraction	The tooth was removed with forceps in the usual manner under local anesthesia.	By a random number generator in the hospital pharmacy	At 1 minute following the initiation of surgery and at the 3-minute mark	0.2% chlorhexidine mouthwash	Streptococcus viridans group and alfa-hemolytic pyogenic streptococci	**CG**: Rinsed vigorously with 10 ml of placebo for 30 seconds and expectorated. Rinsing was repeated 1 min later. **EG**: Rinsed vigorously with 10 ml of 0.2% chlorhexidine hydrochloride for 30 seconds (after the patient was anesthetized) and expectorated. Rinsing was repeated 1 min later	Overall.:CG: 94% EG: 84%	CG: 94%EG: 84%Values from blood samples collected at either the 1-minute and/or 3-minute mark following the initiation of surgery	12 patients were dropped:- 1 patient because of the loss of one of the two blood samples- 11 patients because of problems acquiring or maintaining an intravenous line	Not recorded
Tomás[27]. (2007). Spain. Not specified	CG (n = 53): Control group.EG (n = 53): Chlorhexidine group	The number of teeth to be extracted during the intervention depended on each patient	Dental extraction under general anesthesia (because of mental and behavioral disabilities)	Randomization was based on a single sequence of random assignments (simple randomization) created by applying a computer-generated randomization list.	At baseline (before performing the dental manipulation, but after endotracheal intubation), 30 seconds, 15 minutes, and 1 hour after the final dental extraction (after finishing the surgical procedure)	0.2% chlorhexidine mouthwash	Streptococcus species, particularly viridans group streptococci	**Control group**: Received no chlorhexidine prophylaxis before the dental manipulation.**Chlorhexidine group**: Had their mouths filled with a 0.2% chlorhexidine digluconate solution for 30 seconds before the dental manipulation	At baseline: CG: 9%, EG: 8% At 30 seconds: CG: 96%, EG: 79% At 15 min: CG: 64%, EG: 30%At 1 hr:, CG: 20%, EG: 2%	CG: 96% EG: 79%Values from blood samples collected at 30 seconds after the extraction	19 patients were excluded before randomization:- 12 due to the use of antibiotics in the 3 months before the study- 2 due to the use of oral antiseptics- 2 due to a disease that could predispose the patient to infections or bleeding	Not recorded
Tuna[10]. (2012). Turkey. Not specified	PI group (n = 12): Povidone iodine group. CHX group (n = 12): Chlorhexidine group. C Group (n = 10): Control group	An impacted mandibular third molar	With local anesthesia and under conditions considered aseptic. The third molars were exposed by elevating a buccal mucoperiosteal flap. Osteotomies were implemented by using handpieces under sterile saline irrigation	Patients were randomly allocated into three groups via drawing lots by the same blinded researcher	At baseline (preoperatively, before the injection of local anesthesia), 1 and 15 minutes after the completion of the extraction	7.5% povidone iodine mouth rinse. 0.2% chlorhexidine mouth rinse	Streptococcus viridans; 38% were S. anginosus, 13% were S. salivarius and 13% S. mitis	**Group 1**: Rinsed the mouth with 15 ml 7.5% povidone iodine mouth rinse for 1 minute following the initial blood collection. **Group 2**: Rinsed the mouth with 15 ml 0.2% chlorhexidine mouth rinse for 1 minute following the blood collection. **Group 3**: Rinsed the mouth with 0.9% NaCl (sterile saline) solution	At 1 min: .CG: 40%, CHX group 25%, PI group 33%. At 15 min: . CG: 30%, CHX group 17%, PI group 0%	CG: 40% CHX group: 25%Values from blood samples collected at 1 minute after the extraction	4 patients were excluded:- 2 from control group due to injury of the venous pathway during the insertion of the angiocath. - 2 from chlorhexidine group due to the presence of bacteremia discovered in the preoperative blood culture	Not recorded
Maharaj[26] (2012). South Africa Not specified	Group A (n = 40): Control group.Group B (n = 40): Chlorhexidine group .Group C (n = 40): Amoxicillin group.Group D (n = 40): Clindamycin group	Only one tooth	The same dental surgeon performed the procedure using dental forceps. No surgical procedures were used in any patient	Using a computer-generated randomization table	8–10 ml of blood was drawn three minutes after the extraction in each patient	0.2% chlorhexidine rinse	Viridans streptococci	**Group A**: No therapy prior to dental extraction.**Group B**: Rinsed their mouths vigorously with 10 ml of 0.2% chlorhexidine for one minute and expectorated, and this procedure was repeated one minute later. **Group C**: Took 3 g amoxicillin orally one hour prior to the dental extraction.**Group D**: Took 600 mg clindamycin orally one hour prior to the dental extraction.	Group A: 35%. Group B: 40%. Group C: 7.5%. Group D: 20%	Group A: 35%. Group B: 40%Values from blood samples collected at 3 minutes after the extraction	-	Not recorded
Duvall[11] (2013). USA. June 2011-December 2011	CG (n = 10): Control group. CHX group (n = 10): Rinse group.AMOX group (n = 10): Antibiotic group	4 third molars: tooth #1, tooth #32, tooth #16 and tooth #17	Under conscious sedation and local anesthetic	Via a computer-generated model	At baseline, 1.5 min following initiation of the mucogingival flap #32, 1.5 min following initiation of the mucogingival flap #17, and 10 min following initiation of the mucogingival flap #17	0.12% chlorhexidine rinse	Viridans group streptococci	**Control group**: Placebo capsule 1 hour prior to the procedure + placebo rinse administered immediately prior to conscious sedation medication administration (15 ml for 1 min and expectorated). **CHX group**: Placebo capsule 1 hour to the procedure + 15 ml of chlorhexidine rinse for 1 min. **AMOX group**: 2 g amoxicillin 1 hour prior to the procedure + 15 ml of placebo rinse for 1 min	Overall:CG: 50%CHX group: 60%.AMOX group: 40%	CG: 50%. CHX group: 60%. Values from positive cultures at least one of the time points in which blood samples are collected	7 subjects were not included in the study due to technical issues involving complications during blood draws and/or unavailable microbiological laboratory support	Not recorded
Ugwumba[25] (2014). Nigeria. November 2012 and June 2013	CG (n = 42): Control group.Test group (n = 48): Chlorhexidine group	One or more molar teeth	Under local anesthesia using extraction forceps and/or an elevator	Using computer generated groups placed in white opaque envelopes	At baseline, 1 min and 15 min after the last tooth extraction	0.2% chlorhexidine mouthwash	Staphylococcus aureus	**CG group**: sterile water mouthwash administered for 1 min before any dental manipulation. **Test group**: 0.2% chlorhexidine mouthwash administered for 1 min before any dental manipulation	Overall:CG group: 52.4%.Test group: 27.1%	CG: 33.3%Test group: 17%.Values from blood samples collected at 1 minute after the last tooth extraction	11 subjects were excluded from the analysis of prevalence of bacteremia due to have positive baseline blood cultures (5 in control group and 6 in chlorhexidine group)	Not recorded
Barbosa[24] (2015). Portugal. 2010 and 2012	CG (n = 52): Control groupCHX-MW group (n = 50): Chlorhexidine mouthwash group. CHX-MW/SUB_IR group (n = 51): Chlorhexidine mouthwash and subgingival irrigation. CHX-MW/SUPRA_IR group (n = 48): Chlorhexidine mouthwash and supragingival irrigation	A simple and single-tooth extraction	Under local anesthesia by the same calibrated clinician	Using the closed envelope technique	At baseline, 30 seconds after performing the mouthwash and the subgingival or supragingival irrigation, and at 30 seconds and 15 min after completion of the tooth extraction	0.2% chlorhexidine mouthwash	Streptococci (particularly the S. viridans)	**CG group**: no prophylactic regimen**CHX-MW group**: performed a mouthwash with 0.2% chlorhexidine (10 ml for 1 min) before the tooth extraction. **CHX-MW/SUB_IR group**: performed a mouthwash with 0.2% chlorhexidine (10 ml for 1 min) + subgingival irrigation with 1% chlorhexidine (1.8 ml for 1 min) on the tooth to be extracted. **CHX-MW/SUPRA_IR group**: performed a mouthwash with 0.2% chlorhexidine (10 ml for 1 min) + supragingival irrigation with 1% chlorhexidine (10 ml for 1 min) on the tooth to be extracted	At 30 seconds after tooth extraction:. CG group: 52%CHX-MW group: 50%CHX-MW/SUB_IR group: 55%. CHX-MW/SUPRA_IR group: 50%	CG group: 52%.CHX-MW group: 50%Values from blood samples collected at 30 seconds after completion of the tooth extraction	32 patients were excluded before randomization:- 20 due to not meeting inclusion criteria-12 due to declining to participateLost to follow-up = 5 patients:- 2 in the CHX-MW group- 3 in the CHX-MW/SUPRA_IR group	Not recorded

* The bacteremia data selected to generate the database used in the meta-analysis were from blood samples collected within the first 10 minutes after the completion of the extraction or the initiation of surgery (according to the study design)

- Adverse reactions were not recorded in any study

- There were no split-mouth designs

### Risk of bias within studies

Table 2 illustrates the estimated risk of bias in each of the studies.

**Table 2:** Oxford quality scale.

	Randomized?	Randomized appropriate?	Double-blind?	Blind appropriate?	Withdrawals listed?	TOTAL
Rechmann[29]. (1989)	1	0	1	0	0	2
Lockhart[28]. (1996)	1	1	1	1	1	5
Tomás[27]. (2007)	1	1	0	0	1	3
Tuna[10]. (2012)	1	1	0	0	1	3
Maharaj[26]. (2012)	1	1	0	0	0	2
DuVall[11]. (2013)	1	1	0	0	1	3
Ugwumba[25]. (2014)	1	1	0	0	1	3
Barbosa[24]. (2015)	1	1	1	0	1	4

Despite the identification of potential sources of bias, none of the studies were excluded for this reason.

### Results of individual studies

**Fig 2:**
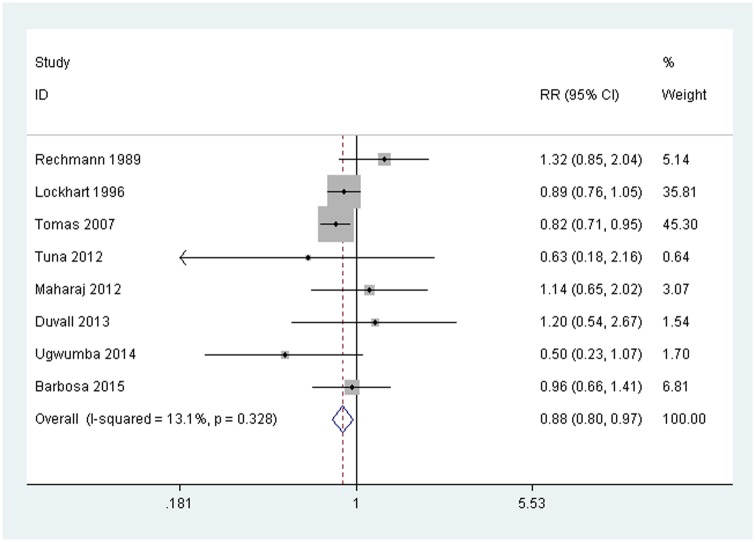
Forest plot. Relative risks (RR) for the effect of treatment with chlorhexidine on the incidence of bacteremia compared to control groups. Fixed-effect inverse variance weighted meta-analysis.

The forest plot “Fig 2” is a graphical representation of the relative risks (RRs) and 95% confidence interval (Cl) estimates based on the samples in each of the studies, together with their relative weights.

### Synthesis of results

#### Efficacy analysis

The quantitative analysis included 523 patients (267 in the experimental group, in which 145 cases of bacteremia were recorded, and 256 in the control group, in which 156 cases of bacteremia occurred). The overall RR was 0.882, with a 95% CI of 0.799 to 0.975; this was statistically significant (p = 0.014). The preventable faction was 0.118, indicating that the percentage of bacteremia cases that can be prevented if a population is exposed to chlorhexidine is 12%.

Taking into account that Oxford Scores below 3 are considered to indicate poor quality clinical trials, we performed a meta-analysis without including the two studies with scores of below 3 to test whether evidence of the benefit of using oral chlorhexidine remained. We have also excluded those trials where blood collection prior to the procedure (baseline) was not record, in order to exclude potential patients who presented subclinical bacteremia.

Five studies were included: Tomas 2007, Tuna 2012, Duval 2013, Uguwmba 2014 y Barbosa 2015. 340 participants were selected, 173 in the experimental group and 167 in the control group. The overall RR, with a fixed effects model with weights calculated using theMantel-Haenszel method was 0.822 (95% confidence interval 0.693 to 0.975; p = 0.024), (heterogeneity I^2^ = 0,0%, and p = 0.503). Excluding these 3 studies we can conclude that the benefit of using oral chlorhexidine remained.

#### Heterogeneity analysis

**Fig 3:**
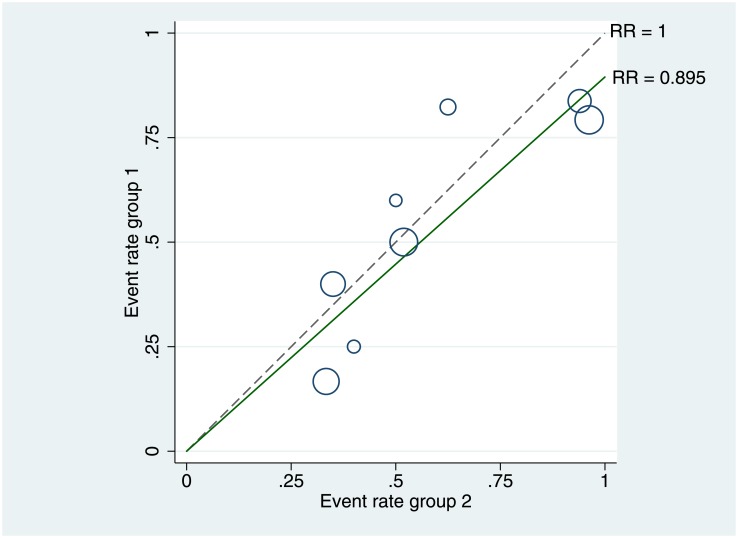
L’Abbé plot of bacteremia rates in the chlorhexidine and control groups. The symbol size represents the sample size. The dotted line represents the no-effect line with identical rates in both groups. The solid line corresponds to a pooled relative risk of 0.895. (RR) = Relative risks.

The Q statistic was 8.053, and I^2^ was 13.074% (p = 0.328), supporting the assumption of homogeneity among the studies. Furthermore, there were no signs of heterogeneity in the L’Abbé plot “Fig 3”; all circles were grouped closely together, regardless of their size and baseline risk.

**Fig 4:**
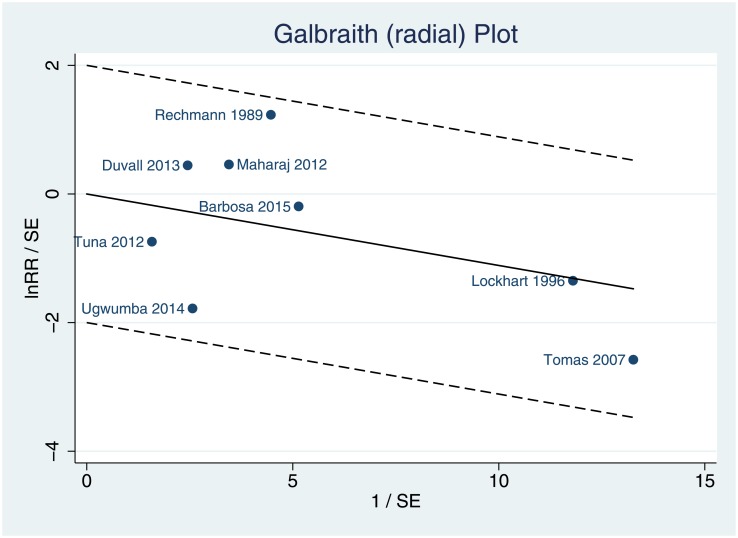
Galbraith (radial) plot for trials that used chlorhexidine for the prevention of post-extraction bacteremia. It can be observed that all studies are within the confidence intervals. (RR) = Relative risks.

The radial Galbraith plot “Fig 4” permits the indirect assessment of heterogeneity.

#### Effectiveness analysis

The overall NNT was estimated to be 16 (IC 95% 7-Infinity). The NNT of each study is shown in Table 3.

**Table 3:** Number needed to treat (NNT) or number needed to harm (NNH) of each study and the global NNT.

Author. (year)	NNT CI 95%/NNH CI 95%
Rechmann[29] (1989)	Number needed to harm: For every 5 patients treated with chlorhexidine, 1 adverse event will occur beyond those that would have occurred under control conditions. 95% confidence interval: [2- Infinity]
Lockhart[28]. (1996)	Number needed to treat: 10 patients must be treated with chlorhexidine to prevent 1 adverse event that would have occurred under control conditions. 95% confidence interval: [5-Infinity]
Tomás[27]. (2007)	Number needed to treat: 6 patients must be treated with chlorhexidine to prevent 1 adverse event that would have occurred under control conditions. 95% confidence interval: [4–21]
Tuna[10]. (2012)	Number needed to treat: 7 patients must be treated with chlorhexidine to prevent 1 adverse event that would have occurred under control conditions. 95% confidence interval: [2-Infinity]
Maharaj[26]. (2012)	Number needed to harm: For every 20 patients treated with chlorhexidine, 1 adverse event will occur beyond those that would have occurred under control conditions. 95% confidence interval: [4- Infinity]
DuVall[11]. (2013)	Number needed to harm: For every 10 patients treated with chlorhexidine, 1 adverse event will occur beyond those that would have occurred under control conditions. 95% confidence interval: [2- Infinity]
Ugwumba[25].(2014)	Number needed to treat: 6 patients must be treated with chlorhexidine to prevent 1 adverse event that would have occurred under control conditions. 95% confidence interval: [3- Infinity]
Barbosa[24]-(2015)	Number needed to treat: 52 patients must be treated with chlorhexidine to prevent 1 adverse event that would have occurred under control conditions. 95% confidence interval: [5- Infinity]
	GLOBAL: Number needed to treat: 16 patients must be treated with chlorhexidine to prevent 1 adverse event that would have occurred under control conditions. 95% confidence interval: [7-Infinity]

### Risk of bias across studies

**Fig 5:**
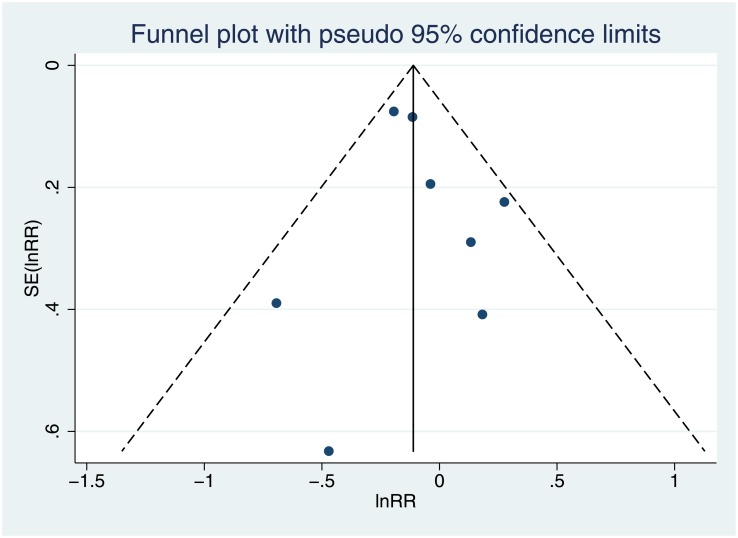
Funnel plot of standard error (in (relative risk)) against in(relative risk).

The funnel plot “Fig 5” may suggest some publication bias because the dispersion pattern of the points was not symmetrical in relation to RR = 0.882.

To avoid a subjective interpretation, we performed a quantitative analysis. Begg´s method suggested the absence of publication bias (Kendall Tau equals 0.071; p = 0.902). The most sensitive method, Egger, also suggested the absence of publication bias (the value of the intercept was 0.384, which was not significant (p = 0.586)), and Macaskill’s procedure yielded a slope that was close to zero and non-significant p = 0.536, indicating a lack of publication bias. Finally, with Rosenthal’s method, we estimated that it would be necessary to add as many as 22 non-significant studies to cause the results of this metal-analysis to become non-significant.

### Additional analysis

#### Cumulative analysis

**Fig 6:**
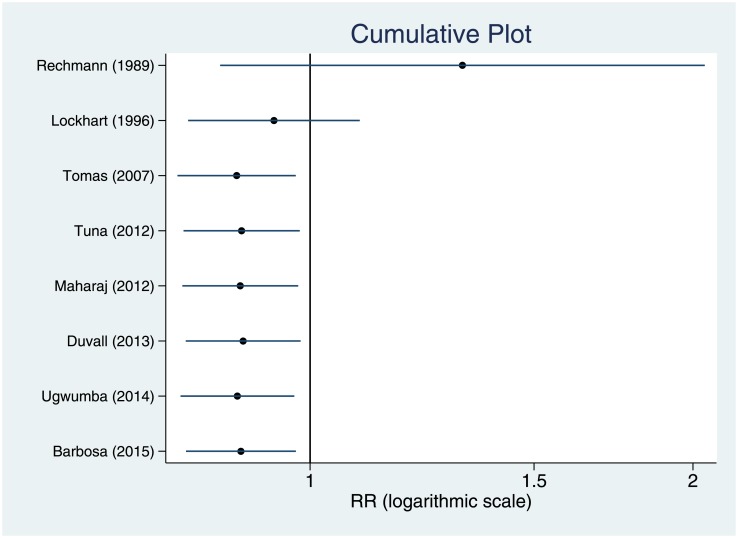
Cumulative meta-analysis by year of publication. Cumulative plot showing the time tendency of relative risk for the incidence of bacteremia.

“Fig 6” shows the evolution of the 95% CI of the weighted RR estimated in the cumulative meta-analysis by year of publication, i.e., as we added studies chronologically to the analysis.

#### Analysis of influence

**Fig 7:**
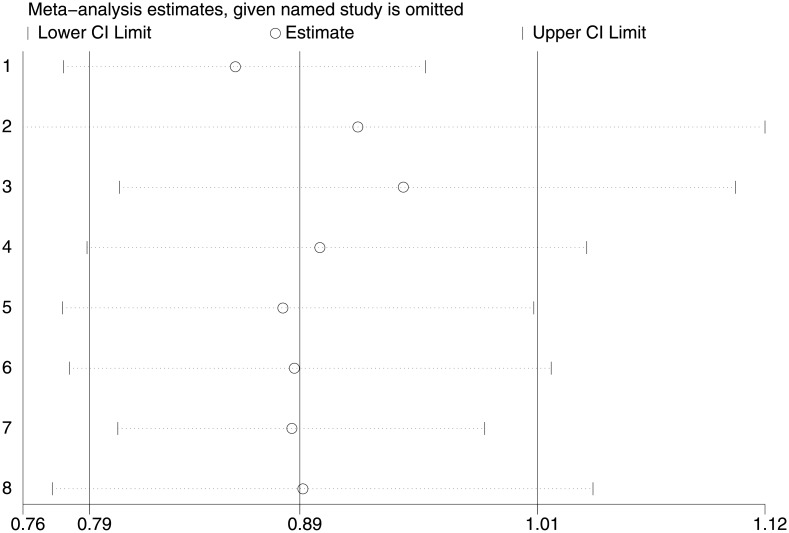
Analysis of influence or sensitivity analysis. Sensitivity analysis showing the influence of individual studies on the summary relative risk. The results are computed by the sequential removal of individual studies. The two ends of every dotted line represent the 95% confidence interval.

The result of the analysis of influence “Fig 7” shows that the estimated value of RR did not change significantly by eliminating a study each time.

#### Adverse reactions

Adverse reactions were not recorded in any study (Table 1).

## Discussion

Due to the existing controversy regarding the use of antiseptics in the prevention of post-dental extraction bacteremia, the exploration of the efficacy of chlorhexidine in the prevention was of interest. To our knowledge, this is the first systematic review and meta-analysis to assess the efficacy of antiseptics for preventing bacteremia associated with tooth extractions.

In summary, we found that antiseptics could reduce the incidence of bacteremia. Our study, which included 523 patients from 8 RCTs, indicates that pre-extraction chlorhexidine treatment significantly reduces bacteremia cases by 12%.

This meta-analysis combines data across studies to estimate antiseptic treatment effect with more precision than in a single study. The main limitation of this meta-analysis is the small number of included studies and their small patient population, which could influence the results. More randomized trials would be required to increase the accuracy of the results. We should also consider that only one of the primary studies [27] reported a statistically significant result and that the overall result can only be generalized to populations with the same characteristics as the populations included in the studies. The sample of patients in this single study had the greatest weight within the meta-analysis, approximately 40%.

The quality of the included studies can also be a handicap in the results of our meta-analysis. The methodological quality of randomized controlled trials (RCTs) is commonly evaluated in order to assess the risk of biased estimates of treatment effects. A commonly used three-item, five-point quality scale was used to rate the quality of the trials [13]. The minimum score possible for inclusion of a study in the review was 2 (one point each for randomization and double-blinding). The maximum score possible was 5 (2 points for descriptions of randomization, 2 points for descriptions of double-blinding, and 1 point for descriptions of withdrawals). Scale limitations should be taken into consideration, and the information provided by scales should be interpreted with caution. In this meta-analysis, two studies had an Oxford Quality Scale score of less than 3, indicating that the quality of these studies may have been low. The decision to include all studies regardless of the date of publication or score obtained in the Oxford Scale means that not all included RCTs have followed the recommendations of the CONSORT statement (Consolidated Standards of Reporting Trials). It is important that the information supplied in publications of clinical trials is sufficient and accurate. The exclusion of RCTs with a value lower than 3 in the Oxford scale [26, 29] and the exclusion of articles [26, 28] which do not clearly establish the level of basal bacteremia showed that the benefit of using oral chlorhexidine remained.

The treatment guidelines used in the different trials included in the meta-analysis differ slightly, although the use of a 0.20% chlorhexidine mouthwash for 1 minute prior to extraction is the most common recommendation. However, the time of blood sample extraction was different in each study. In some cases, there was only one sample, while in others, serial blood sample extractions were done post-extraction. These discrepancies should be taken into account when analyzing the results and were considered when the response variable had to be defined in our study. Bacteremia follows an upward trend post-extraction, peaks at 1–5 minutes, and then drops for up to 15 minutes [30, 31], which indicates that the post-extraction time when the sample is taken is essential. Therefore, we decided to exclude the values of all measurements performed more than 10 minutes post-extraction.

Although it was not our objective to analyze the bacteria involved in bacteremia, our meta-analysis confirmed that the bacteria most commonly isolated in the cultures was the *viridans group streptococci*, except for the trial performed by Ugwumba [25] in which the most common bacteria was *Staphylococcus aureus*. Over 700 types of bacteria have been identified in the oral cavity, and over 170 in post-extraction blood cultures; however, the bacteria directly related to IE pathogenesis is the *viridans group streptococci* [32]. Therefore, any IE prevention protocol should be aimed at preventing infection by this streptococcus.

It is remarkable that no adverse reactions were recorded in any of the studies. We propose that this variable be included in all trials. Perhaps this was not recorded since none occurred, and if this is case, then it must be explicitly included as a result in each study.

It is well-known that tooth extraction is associated with peaks of bacteremia, although most of these peaks do not progress to a clinical infection. It would be important to demonstrate that a disinfectant, possibly chlorhexidine, reduces the risk of clinical infection after dental extractions, but no articles have been published on this subject.

The ultimate purpose of this revision is to help the clinician make a decision in daily practice. With the results obtained from combining the few quality studies available, we can state there is evidence to support the efficacy of using chlorhexidine pre-extraction to reduce bacteremia. The use of chlorhexidine only reduces post-extraction bacteremia risk by 12%. The number necessary to treat to prevent just 1 case of bacteremia is 16. Nevertheless, bearing in mind that it is an economic and easy to use antiseptic with no evidence of adverse reactions, its use can be recommended despite its low efficacy. More studies on this topic are required to achieve accurate conclusive results to enable decision making based on a sufficient number of quality clinical trials.

## Supporting information

S1 Table PRISMA checklist. (DOC)

S1 File Meta-analysis data set. (ODT)
