# Supplementary material for: Does chlorhexidine reduce bacteremia following tooth extraction? A systematic review and meta-analysis
Source: PLoS One. 2018 Apr 23;13(4):e0195592. doi: 10.1371/journal.pone.0195592 (PMC5912716; doi:10.1371/journal.pone.0195592)
Supplement: Supplementary file 2 [file pone.0195592.s002.odt]

I[ar Arteagoitia](https://www.protocols.io/researchers/iciar-arteagoitia)

- 1

  - Data sources: Pubmed, Cochrane, Web of Science, Science Direct, Scopus, and Ovid MD. Searched until 30th April 2017. (chlorhexidine) AND (bacteremia OR bacteraemia) AND (extraction OR removal) were used as key words in a free-text search. Meeting Abstracts published were searched. References of each article were reviewed. We only included randomized controlled clinical trials. There were no restrictions regarding language or date of publication. The outcome measure was set on the incidence of the bacteremia measure within the first ten minutes post-extraction. Two reviewers independently undertook the risk of bias assessment and data extraction.

Out of 18 studies, eight eligible trials (with 523 participants were selected, 267 in the experimental group and 256 in the control group)

 

| study | date | a1 | n1 | a0 | n0 |
| --- | --- | --- | --- | --- | --- |
| Rechmann 1989 | 01/07/1989 | 14 | 17 | 10 | 16 |
| Lockhart 1996 | 01/07/1996 | 31 | 37 | 31 | 33 |
| Tomas 2007 | 01/07/2007 | 42 | 53 | 51 | 53 |
| Tuna 2012 | 01/07/2012 | 3 | 12 | 4 | 10 |
| Maharaj 2012 | 01/07/2012 | 16 | 40 | 14 | 40 |
| Duvall 2013 | 01/07/2013 | 6 | 10 | 5 | 10 |
| Ugwumba 2014 | 01/07/2014 | 8 | 48 | 14 | 42 |
| Barbosa 2015 | 01/07/2015 | 25 | 50 | 27 | 52 |

 

The following data were extracted for each study included in the meta-analysis: primary author, year of publication, location, recruitment period, number of patients randomized to each study group, details of the surgical procedure and the randomization method, time of collection of blood samples for culture, antiseptic used (including the concentration and dosage form) and the regimen ((dose and guideline),of administration of the antiseptic in each study group, the most bacteria most often identified in the cultures, the incidence of bacteremia according to the time of assessment and the bacteremia data selected for our meta-analysis. Details about adverse reactions, exclusions, withdrawals and losses were also recorded.

A fixed-effect inverse variance weighted meta-analysis was conducted.

Risk ratio= 0.882 (95% confidence interval 0.799 to 0.975; p= 0.014), heterogeneity I²=13.07%, and p=0.33. The number needed to treat was 16 (95%

- 2
